# Effect of Licochalcone-A Combined with *Rab23* Gene on Proliferation of Glioma U251 Cells

**DOI:** 10.1155/2022/9299442

**Published:** 2022-04-22

**Authors:** Yindong Mu, Jianjiang Dong, Hong Cui, Jiangping Hu, Jun Liang, Lei Yan

**Affiliations:** ^1^Department of Histology and Embryology, Mudanjiang Medical University, Mudanjiang 157011, China; ^2^Department of Pharmacy, Hongqi Hospital Affiliated to Mudanjiang Medical University, Mudanjiang 157011, China; ^3^Stem Cell Institute, Mudanjiang Medical University, Mudanjiang 157011, China

## Abstract

This research aimed to explore the effect of Licochalcone-A (LCA) combined with *Rab23* gene on the proliferation, migration, and invasion of glioma U251 cells through the Wnt/*β*-catenin signaling pathway. The glioma U251 cell line was taken as the research object, and the Rab23 overexpression plasmid was constructed. According to the treatment method, U251 cells were rolled into blank control group (BC), Rab23 overexpression plasmid transfection group (Rab23), 25 *μ*mol·L^−1^ LCA treatment group (LCA), and Rab23 overexpression plasmid transfection combined with 25 *μ*mol·L^−1^ LCA treatment group (Rab23 + LCA). Subsequently, the ability of cell proliferation, migration, and invasion of each group was detected by methyl thiazolyl tetrazolium (MTT) assay, scratch healing test, and Transwell cell invasion test, respectively. Western blot was implemented to detect the expression differences of cell proliferation antigen Ki-67, apoptosis-related proteins Bcl-2 and Bax, and Wnt/*β*-catenin pathway-related proteins *β*-catenin, glycogen synthase kinase-3 (GSK3*β*), Axin2, and c-myc. The results showed the successful construction of Rab23 overexpression and stable transfection U251 cell line. After grouping and treatments, the cell proliferation, migration, and invasion ability of the Rab23 group, LCA group, and Rab23 + LCA group was substantially reduced relative to BC group (*P* < 0.05). In addition, the cell proliferation, migration, and invasion ability of Rab23 + LCA group decreased relatively more significantly. The expression levels of Ki-67, Bcl-2, *β*-catenin, and c-myc in the Rab23, LCA, and Rab23 + LCA groups were greatly lower versus those of BC group. Moreover, the protein expression levels of Bax, GSK3*β*, and Axin2 were considerably increased (*P* < 0.05), while the expression of protein in Rab23 + LCA group increased notably. These findings indicate that LCA combined with *Rab23* gene can inhibit the proliferation, migration, and invasion of glioma U251 cells through the Wnt/*β*-catenin signaling and can promote cell apoptosis.

## 1. Introduction

At present, the mortality rate of malignant tumors accounts for about 24% of all causes of death, and the incidence and mortality rate of nervous system tumors account for the top ten of all malignant tumors. The pathogenesis of primary central nervous system tumors is not clear and has the characteristics of strong invasion, high recurrence rate, and high mortality, among which glioma accounts for more than 50% [1]. The current standard clinical treatment of glioma is surgery combined with chemotherapy or radiotherapy. Glioma is highly invasive, and the prognosis of patients is very poor, with the recurrence rate reaching 100%, while the 5-year survival rate is less than 5% [2]. Multidisciplinary treatment, including surgery, radiotherapy, and chemotherapy, is the standard treatment for glioma, but the survival time of most patients after treatment does not exceed two years [3]. Therefore, the development of new drugs for killing glioma cells is of great significance for reducing the recurrence rate and mortality after treatment and improving the quality of life of patients and the prognosis.

Chinese herbal medicine is one of the preferred methods of modern medicine to treat diseases, and the antitumor activity and the advantages of low side effects of Chinese herbal medicine have also received extensive attention from people. Licochalcone-A (LCA) extracted from licorice is a kind of natural active brass, and it has antitumor activity [4]. At present, many studies confirmed that LCA had excellent anticancer effects, such as in liver cancer [5], gastric cancer [6], breast cancer [7], and bladder cancer [8]. The process of tumor cell invasion and metastasis is regulated by multiple genes and steps. *Rab23* gene is a member of the Ras oncogene family. Studies confirmed that the *Rab23* gene played an imperative role in the occurrence and progression of tumors [9]. In addition, the overexpression of *Rab23* gene can inhibit the proliferation activity of glioma cells [10]. In recent years, studies verified that the disorder and excessive activation of the Wnt signaling pathway can participate in the biological processes of glioma cell proliferation, migration, and invasion [11].

However, the mechanism of *Rab23* gene combined with LCA in the invasion and metastasis of glioma remains to be explored. In this research, glioma U251 cells were deemed as the research object, and the effects on cell proliferation, migration, and invasion were explored through overexpression of Rab23 and LCA treatment. Then, the mechanism of Wnt/*β*-catenin pathway in the proliferation, migration, and invasion of glioma U251 cells was preliminarily explored.

## 2. Materials and Methods

### 2.1. Experimental Materials

The glioma cell line U251 was purchased from ATCC, USA. Fetal bovine serum, high glucose DMEM, penicillin mixture, and trypsin were all purchased from Gibco, USA. LCA powder (molecular formula: C_21_H_22_O_4_; molecular weight: 338.40; purity ≥ 96.0%), dimethyl sulfoxide preparation, and methyl thiazolyl tetrazolium (MTT) kit were purchased from Sigma, USA. A 4% paraformaldehyde was purchased from Invitrogen, USA. Radio immunoprecipitation assay (RIPA) cell lysate was purchased from Beyotime Institute of Biotechnology, China. Bicinchoninic acid (BCA) kit and horseradish peroxidase-labeled goat anti-mouse IgG antibody were purchased from Thermo Company, USA. The ultrasensitive ECL chemiluminescence kit was purchased from Advansta, USA. Mouse anti-human Ki-67, *β*-catenin, GSK3*β*, Axin2, c-myc, Bcl-2, Bax, and GAPDH were all purchased from Cell Signaling, USA. Puromycin was purchased from Merck, USA. Lentiviral Vector Particle was purchased from China Jikai Gene. The cell culture dishes were purchased from Corning Costar, USA.

### 2.2. Experimental Methods

#### 2.2.1. Cultivation of Glioma U251 Cells

Glioma U251 cells were cultured in Dulbecco's modified eagle medium (DMEM) containing 10% fetal bovine serum (FBS) and 100 UL^−1^ penicillin streptomycin, and placed in a 37°C thermostatic cell culture incubator (Thermo Company, USA) containing 5% CO_2_. When the degree of cell fusion reached 80%, the cells were digested with trypsin and passed at a rate of 1 : 4. In this study, 2–13 generations of cells with the best culture activity were used for subsequent experiments.

#### 2.2.2. Grouping and Transfection of Glioma U251 Cells

The glioma U251 cells were rolled into blank control group (BC), Rab23 transfection group (Rab23), LCA group (LCA), and Rab23 + LCA group (Rab23 + LCA). The cells in Rab23 group were transfected with Rab23 overexpression plasmid; cells in LCA group were treated with 25 *μ*mol·L^−1^ LCA; and the cells in the Rab23 + LCA group were transfected with Rab23 overexpression plasmid and 25 *μ*mol·L^−1^ LCA.

The cells were transfected according to the instructions of Lentiviral Vector Particle. Glioma U251 cells were inoculated into 96-well plates, and 1 × 10^8^ TU·mL^−1^ transfection solution (2 *μ*L virus mixed with 98 *μ*L DMEM containing 10% FBS) was added into each well when the fusion degree reached about 70%. After 12 h of culture, the original medium was discarded, and conventional cell medium was added to culture for 3 days for fluorescence observation. After 7 days of culture, the cells were inoculated into 24-well plates when the fluorescence expression of the cells exceeded 70%. Routine cell culture medium containing 1 mg·L^−1^ puromycin was added for culture for 7 days, to obtain stable overexpression of Rab23 cell lines. Western blot was performed to detect the expression of Rab23 in glioma U251 cell lines transfected with lentivirus.

#### 2.2.3. MTT Assay

The cells were seeded into 96-well cell culture plates at a density of 1 × 10^7^ cells·mL^−1^, the inoculation volume was 100 *μ*L per well, and five replicates were set in each group. When the degree of cell fusion reached about 80%, the cells were passed and grouped. After 48 h of culture, the original medium of cells in each well was removed, and 20 *μ*L MTT working solution was added to each well successively. The cell culture plates were replaced in a 37°C constant temperature cell incubator containing 5% CO_2_ and incubated in dark place for 4 h. Then, the original MTT working fluid of cells in each well was removed, and 150 *μ*L dimethyl sulfoxide reagent was added to each well successively. The MTT working fluid in each well was shaken at low speed for 10 min avoiding light. Finally, the absorbance (OD) of cells in each well was measured at 490 nm using a microplate analyzer (BioTek, USA). The relative viability of cells was calculated according to the equation: (average absorbance in the test group/average absorbance in the blank group) ×100%.

#### 2.2.4. Scratch Healing Test

Cells were seeded in a 6-well cell culture plate with the density of 4 × 10^5^ cells·mL^−1^, and cultured for 24 h. When the degree of cell fusion reached about 80%, the sterilized 100 *μ*L pipettor head was used to scratch the monolayer cells perpendicular to the bottom of the orifice plate. The cells were gently washed with phosphoric acid buffer (PBS) 3 times. After transfection and administration, new cell medium was added to each well for further culture for 24 h. A microscope (Nikon, Japan) was employed to take pictures of monolayer cells with different field of view and the width of the scratches is measured. The cell scratch healing rate was calculated according to the equation: (scratch width of 0 h – scratch width of 24 h)/scratch width of 0 h × 100%.

#### 2.2.5. Transwell Chamber Cell Invasion Test

Cell concentration was adjusted to 2 × 10^5^ cells·mL^−1^, and transfected and administered, respectively. After 24 h of normal culture, serum-free starvation lasted for 12 h. After trypsin digestion, the cell density was adjusted to 1 × 10^5^ cells·mL^−1^ using serum-free medium. Then, 50 mg·L^−1^ Matrigel 1 : 8 diluent was used to coat the upper compartment surface of the membrane at the bottom of the Transwell chamber, and the solution was fixed in an incubator for 30 min. After this, the residual liquid in the chamber was removed, and 50 *μ*L serum-free culture medium was added to each well, hydrated for 30 min. Then, 200 *μ*L cell suspension was taken and placed in Transwell chamber of 24-well plate. The experiment was repeated for three times in each group. A quantity of 500 *μ*L high glucose DMEM containing 20% FBS was added into the lower chamber, and put into 37°C constant temperature cell incubator containing 5% CO_2_ for 24 h. Three days later, the Transwell chamber was removed, the matrix glue and cells in the chamber were gently wiped with a sterile cotton swab, and 4% paraformaldehyde solution was added to fix the cells for 5 min. Then, the residual liquid was discarded, and 4 g·L^−1^ crystal violet solution was added for staining for 20 min. Then, the cells were washed for 3 times with PBS. Ten fields were randomly selected from each sample. The cells transferred to the subcellular membrane were counted using an inverted microscope (Nikon, Japan) and averaged.

#### 2.2.6. Western Blot

The treated cells of each group were taken, RIPA cell lysate was added, and the supernatant was taken after centrifugation at 12,000 rpm for 20 min. According to the instructions of the BCA kit, the total protein concentration was measured at 570 nm using an enzyme plate analyzer. Then, Western blot analysis was performed on 40 *μ*g protein. After 10% sodium dodecyl sulfate-polyacrylamide gel electrophoresis (SDS-PAGE) gel electrophoresis, transfer blot was performed, and the membrane was sealed overnight with 5% skimmed milk at 4°C. Mouse anti-human Ki-67 (1 : 1000), Bcl-2 (1 : 1000), Bax (1 : 1000), *β*-catenin (1 : 1000), GSK3*β* (1 : 1000), Axin2 (1 : 1000), c-myc (1 : 1000), and GAPDH (1 : 1000) primary antibodies were added, and sealed overnight at 4°C. Then, horseradish peroxidase (HRP) labeled goat anti-rat IgG (1 : 2000) secondary antibody was added and incubated at room temperature for 2 h. According to the instructions of the ECL chemiluminescence kit, the color of protein bands was developed. After exposure, ECL chemiluminescence instrument (Bio-Rad, USA) was utilized for development and fixing. Quantity One was then employed to analyze the gray value of the target protein bands. The relative expression of target protein was calculated according to the equation: gray value of target protein band/gray value of GAPDH band.

### 2.3. Statistical Methods

SPSS 19.0 was employed for statistical analysis. The test data were all expressed as mean plus or minus standard deviation ($\bar{x}\pm s$), and differences were compared by one-way ANOV A analysis. *P* < 0.05 was considered statistically significant.

## 3. Results

### 3.1. Evaluation of Stable Transformation of U251 Cells Overexpressing Rab23

**Figure 1:**
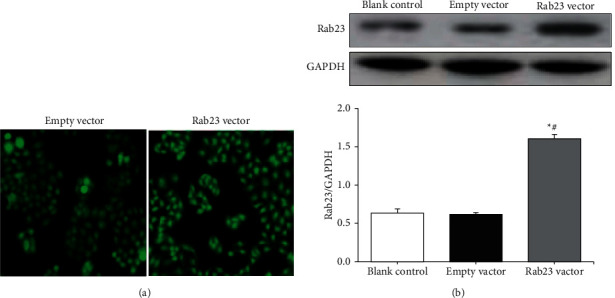
Identification of U251 cells with stable empty vector and overexpressing Rab23. (a) Micrograph of U251 cells (×200); (b) Rab23 protein expression. In contrast to blank control group, ^*∗*^*P* < 0.05; in contrast to empty vector transfection group, ^#^*P* < 0.05.

The fluorescence expression in U251 cells after transfection was observed with an inverted fluorescence microscope.  Figure 1(a) shows that after transfection with the empty vector and the Rab23 overexpression vector, the U251 cells all had green fluorescence expression, and they were evenly distributed in the cytoplasm. Western blot detection results of Rab23 protein expression in cells in Figure 1(b) showed that there was no significant difference in Rab23 protein expression between the blank control group and the transfected empty vector group (*P* > 0.05). In contrast to blank control group and the transfected empty vector group, the Rab23 protein expression level in the cells of the Rab23 overexpression plasmid transfection group was remarkably increased (*P* < 0.05).

### 3.2. Detection of U251 Cell Proliferation Inhibition by MTT Assay

**Figure 2:**
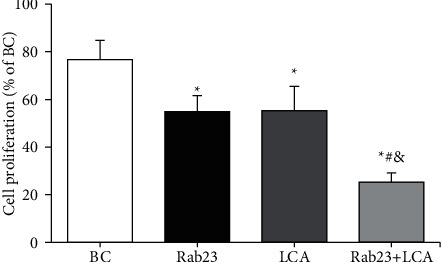
A comparison of U251 cell proliferation activity in each group. ^*∗*^*P* < 0.05 versus BC group; ^#^*P* < 0.05 versus Rab23 group; ^&^*P* < 0.05 versus LCA group.

MTT assay was adopted to detect the differences in cell proliferation levels of U251 cells in different treatment groups after 48 hours of grouping treatment.  Figure 2 presents that the cell proliferation activity of the Rab23 group, the LCA group, and the Rab23 + LCA group was greatly reduced compared to that of the BC group (*P* < 0.05). The cell proliferation activity of the Rab23 + LCA group was considerably reduced relative to that of Rab23 group and the LCA group (*P* < 0.05), while there was no obvious difference in the cell proliferation activity between the Rab23 group and the LCA group (*P* > 0.05).

### 3.3. Scratch Healing Test to Detect the Migration Ability of U251 Cells

**Figure 3:**
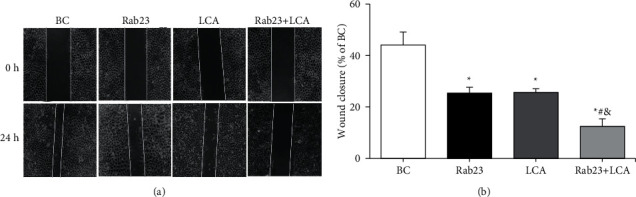
A comparison of scratch healing degree of U251 cells in each group. (a) Microscopic image of cell scratch (×100); (b) statistical graph of cell scratch healing. ^*∗*^*P* < 0.05 versus BC group; ^#^*P* < 0.05 versus Rab23 group; ^&^*P* < 0.05 versus LCA group.

The scratch healing test was performed to evaluate the migration ability of U251 cells in different treatment groups, and the results are shown in Figure 3. From Figure 3(a), there was no substantial difference in the scratch healing degree among the four groups of cells at 0 h of the scratch. After 24 h of scratching, the scratches in the BC group healed significantly, the scratches in the Rab23 group and the LCA group were obvious, and the scratches in the Rab23 + LCA group were more obvious.  Figure 3(b) shows that compared to the BC group, the healing of the scratches of the cells in the Rab23 group, the LCA group, and the Rab23 + LCA group was notably reduced (*P* < 0.05). In contrast to the Rab23 group and the LCA group, the scratch healing of the cells in the Rab23 + LCA group was remarkably reduced (*P* < 0.05), while there was no great difference in the scratch healing of the cells between the Rab23 group and the LCA group (*P* > 0.05).

### 3.4. Transwell Chamber Test to Evaluate Invasion Ability of U251 Cells

**Figure 4:**
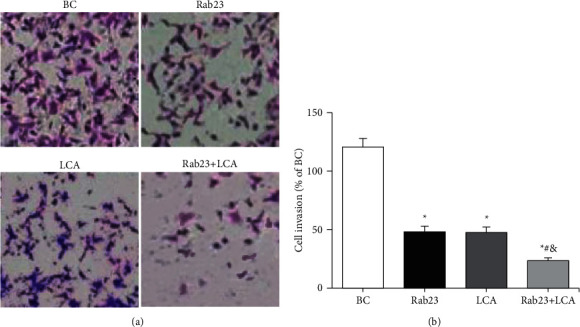
A comparison of U251 cell invasion levels in each group. (a) Cell invasion staining map (×100); (b) cell invasion level statistics. ^*∗*^*P* < 0.05 versus BC group; ^#^*P* < 0.05 versus Rab23 group; ^&^*P* < 0.05 versus LCA group.

The Transwell chamber test was implemented to evaluate the invasion ability of U251 cells in different treatment groups, and the results are shown in Figure 4.  Figure 4(a) shows that the cell invasion staining of the Rab23 group, the LCA group, and the Rab23 + LCA group was obviously less than that of the BC group, and the cell invasion staining of the Rab23 + LCA group was the least. The number of positive U251 cells stained was counted.  Figure 4(b) shows that compared with the BC group, the cell invasion level of the Rab23 group, the LCA group, and the Rab23 + LCA group was substantially reduced (*P* < 0.05). The cell invasion level of the Rab23 + LCA group was evidently inferior to that of Rab23 group and the LCA group (*P* < 0.05), while the Rab23 group and the LCA group had no remarkable difference in the cell invasion level (*P* > 0.05).

### 3.5. Western Blot Detection of Ki-67 and Apoptosis-Related Protein Expression in U251 Cells

**Figure 5:**
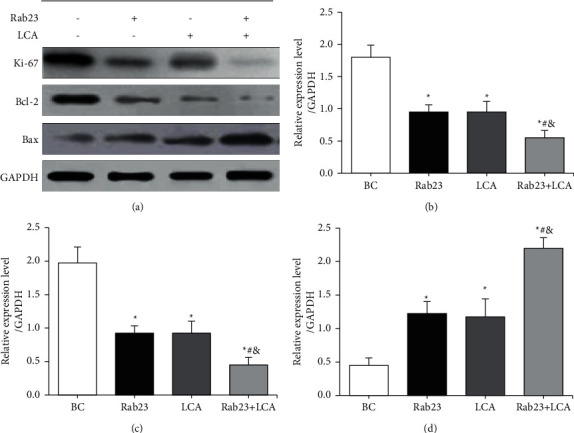
The differences in the expression of proliferation antigens and apoptosis proteins of U251 cells in each group. (a) Western blot; (b) relative expression of Ki-67 protein; (c) relative expression statistics chart of Bcl-2 protein; (d) relative expression statistics chart of Bax protein. ^*∗*^*P* < 0.05 versus BC group; ^#^*P* < 0.05 versus Rab23 group; ^&^*P* < 0.05 versus LCA group.

Western blot was adopted to detect the differences in the expression of Ki-67 antigen and apoptosis-related proteins Bcl-2 and Bax in U251 cells in different treatment groups. The results are shown in Figure 5.  Figure 5(a) shows that the expression of Ki-67 and Bcl-2 protein in the Rab23 group, the LCA group, and the Rab23 + LCA group were significantly reduced relative to those of BC group, while the Bax protein expression was significantly increased. The gray value difference of target protein in each group was counted. From Figures 5(b), 5(c), and 5(d), the expression of Ki-67 and Bcl-2 protein in the Rab23 group, the LCA group, and the Rab23 + LCA group were notably reduced in contrast to BC group, while the Bax protein expression was greatly increased (*P* < 0.05). Compared with the Rab23 group and the LCA group, the expression of Ki-67 and Bcl-2 protein in the Rab23 + LCA group was reduced, and the Bax protein expression was increased greatly (*P* < 0.05). However, there were no significant differences in the expression of Ki-67, Bcl-2, and Bax proteins between Rab23 group and LCA group (*P* > 0.05).

### 3.6. Western Blot Detection of Wnt/*β*-Catenin Related Protein Expression in U251 Cells

**Figure 6:**
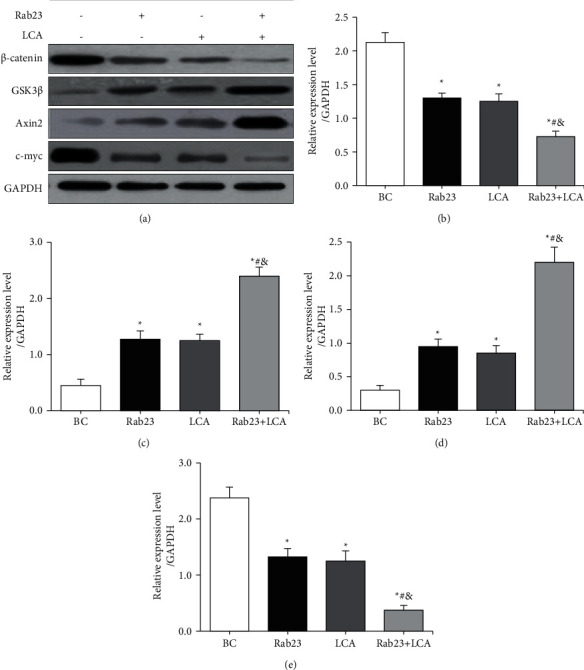
Differences in Wnt/*β*-catenin pathway-related protein expression in U251 cells in each group. (a) Western blot; (b) relative expression of *β*-catenin protein; (c) relative expression of GSK3*β* protein; (d) relative expression of Axin2 protein; (e) statistical graph of relative expression of c-myc protein. ^*∗*^*P* < 0.05 versus BC group; #*P* < 0.05 versus Rab23 group; &*P* < 0.05 versus LCA group.

Western blot was performed to detect differences in the expression of Wnt/*β*-catenin signaling-related proteins *β*-catenin, GSK3*β*, Axin2, and c-myc in U251 cells in different treatment groups. The results are shown in Figure 6.  Figure 6(a) shows that, compared with the BC group, the expression of *β*-catenin and c-myc protein in the Rab23 group, the LCA group, and the Rab23 + LCA group was considerably reduced, while the expression of GSK3*β* and Axin2 protein was substantially increased.

The differences in the gray value of the target protein of each group of cells were statistically analyzed. Figures 6(b)–6(d) show that the expression of *β*-catenin and c-myc protein in the Rab23 group, LCA group, and Rab23 + LCA group was greatly lower than that of the BC group (*P* < 0.05). The expression of *β*-catenin and c-myc protein in the Rab23 + LCA group was notably lower than that in the Rab23 group and the LCA group (*P* < 0.05). From Figures 6(c)–6(e), the expression of GSK3*β* and Axin2 protein in the Rab23 group, LCA group, and Rab23 + LCA group was remarkably inferior to that in the BC group (*P* < 0.05). The expression of GSK3*β* and Axin2 protein in the Rab23 + LCA group was evidently lower than that in the Rab23 group and the LCA group (*P* < 0.05). However, there was no significant difference in the expression of *β*-catenin, GSK3*β*, Axin2, and c-myc in the cells of Rab23 group and LCA group (*P* > 0.05).

## 4. Discussion

Glioma is a primary intracranial tumor with high incidence and high degree of malignancy, and the average survival time of patients is relatively short [12]. *Rab23* gene is closely related to the progression of cancer. Studies found that overexpression of Rab23 can inhibit the proliferation activity of cancer cells and promoted cell apoptosis [13]. At present, the standard treatment for glioma is surgery combined with chemotherapy and radiotherapy, but the treatment efficiency is still unsatisfactory. LCA is a Chinese herbal extract with anti-inflammatory, antiviral, and antitumor properties, and studies showed that LCA can inhibit the proliferation activity of various tumor cells [14, 15]. Therefore, the potential molecular mechanisms of the effects of overexpression of Rab23 and LCA on proliferation, migration, and invasion of glioma U251 cells were explored. The results showed that overexpression of Rab23 and LCA treatment could inhibit the proliferation, migration, and invasion ability of human glioma U251 cells. Moreover, the inhibition effect of overexpression of Rab23 combined with LCA on proliferation, migration, and invasion of human glioma U251 cells was significantly superior to that of single treatment group. These results indicated that overexpression of Rab23 and LCA could inhibit the proliferation, migration, and invasion of glioma U251 cells, and the combined treatment had a better effect.

Studies revealed that LCA can activate mitochondrial-dependent endogenous apoptosis, and can downregulate the level of antiapoptotic protein Bcl-2 and upregulate the level of proapoptotic protein Bax [16, 17]. The balance of Bcl-2 and Bax protein expression levels determines the state of cell survival or apoptosis [18]. Therefore, this work further examined the changes in the expression of Bcl-2 and Bax proteins in cells. Western blot results showed that overexpression of Rab23, LCA simple treatment, and combined treatment can reduce the expression level of Bcl-2 protein and can promote the expression level of Bax protein. Among these proteins, the Bcl-2 protein level decreased and the Bax protein level increased obviously in the combined treatment group, indicating that overexpression of Rab23 and LCA can also promote the apoptosis of glioma U251 cells by regulating the expression levels of Bcl-2 and Bax protein, thus playing an antitumor effect.

Wnt/*β*-catenin signaling pathway participates in the regulation of tumor cell growth and differentiation, and activation of Wnt/*β*-catenin signaling pathway can participate in the process and metastasis of glioma cells [19, 20]. Studies revealed that inhibiting the Wnt/*β*-catenin signaling pathway can inhibit the proliferation and migration of glioma cells [21]. *β*-Catenin is a positive regulator of this pathway, and GSK3*β* and Axin can form a “destruction complex” and bind to *β*-catenin [22]. In tumor cells, the Wnt signaling pathway was activated to transfer *β*-catenin, which in turn lead to the activation of downstream target genes c-myc, thus promoting tumor development [23]. Studies suggested that Axin expression was downregulated in glioma cells, and the activation of GSK3*β* can promote the proliferation and differentiation of glioma cells [24, 25]. Western blot results showed that overexpression of Rab23, LCA alone, and combined treatment can downregulate *β*-catenin and c-myc protein levels in glioma U251 cells, which can upregulate GSK3*β* and Axin2 protein levels.

## 5. Conclusion

In summary, the results of this study preliminarily showed that overexpression of Rab23 and LCA can effectively inhibit the proliferation, migration, and invasion of glioma cells through the Wnt/*β*-catenin signaling, and promoted cell apoptosis. In this study, only glioma cells were used as experimental subjects, and the inhibitory effects of LCA on the proliferation, invasion, and migration of glioma cells were verified in vitro. However, whether LCA can prolong survival and other specific mechanisms remain to be explored. Therefore, in future studies, animal glioma models will be further prepared to explore the effects of LCA treatment on the blood–brain barrier, tumor immune inflammatory microenvironment, and survival rate in animal models, thereby comprehensively exploring the effect of LCA treatment on glioma. Meanwhile, the interaction between LCA and *Rab23* gene will be further explored. The effect of overexpression of Rab23 combined with LCA was even obvious. This study can provide a new research direction for the clinical treatment of glioma.

## Data Availability

The data used to support the findings of this study are available from the corresponding author upon request.
